# In Vitro Evaluation of the Effectiveness and pH Variation of Dental Bleaching Gels and Their Effect on Enamel Surface Roughness

**DOI:** 10.3390/dj12120415

**Published:** 2024-12-18

**Authors:** Federica Veneri, Francesco Cavani, Giovanni Bolelli, Vittorio Checchi, Alessia Bizzi, Giacomo Setti, Luigi Generali

**Affiliations:** 1 Department of Surgery, Medicine, Dentistry and Morphological Sciences with Transplant Surgery, Oncology and Regenerative Medicine Relevance (CHIMOMO), University of Modena and Reggio Emilia, 41124 Modena, Italy; federica.veneri@unimore.it (F.V.); 269479@studenti.unimore.it (A.B.); giacomo.setti@unimore.it (G.S.); luigi.generali@unimore.it (L.G.); 2 Department of Biomedical, Metabolic and Neural Sciences, University of Modena and Reggio Emilia, 41124 Modena, Italy; francesco.cavani@unimore.it; 3 Department of Engineering “Enzo Ferrari” (DIEF), University of Modena and Reggio Emilia, 41124 Modena, Italy; giovanni.bolelli@unimore.it

**Keywords:** bleaching, enamel, discoloration, pH, surface roughness

## Abstract

**Objectives**: Potential adverse effects and pH-related effectiveness of bleaching agents have raised some concerns. The aim of this study was to compare three bleaching agents containing hydrogen peroxide (HP) and carbamide peroxide (CP) in terms of whitening effectiveness, pH variation, and changes in enamel surface roughness. **Methods**: After controlled staining with a black tea solution, 42 human enamel specimens underwent bleaching treatment using the following agents: HP 40%; HP 35%; CP 16%. Color changes were evaluated according to the CIEDE2000 system. Gel pH was measured before and after each application. Surface roughness (Sa) was assessed through optical 3D profilometry before and after bleaching treatment. **Results**: The whitening effectiveness was similar for HP 40% and HP 35% while CP 16% had significantly lower results. HP 40% showed a remarkable pH acidification (−0.41), while HP 35% and CP 16% showed a mild increase in pH values (+0.26 and +0.03, respectively), and the differences between HP 40% and HP 35% and between HP 35% and CP 16% were statistically significant. Sa slightly decreased in all groups after bleaching, with no significant differences among them and a significant difference in HP 40% before and after treatment. **Conclusions**: Similar bleaching results were achieved regardless of pH and HP concentration for HP-based agents, while a lower bleaching effect was observed for the less concentrated CP-based agent, as anticipated. Higher HP and greater tendency to pH instability induced more pronounced modifications of surface roughness. This in vitro study suggests that bleaching gels with neutral and stable pH ensure good bleaching effectiveness and are less likely to cause enamel surface changes.

## 1. Introduction

Dental discoloration is one of the most common esthetic concerns in dentistry and is caused primarily by extrinsic staining agents, such as coffee, tea, red wine, smoking, and long-term chlorhexidine use [1,2]. Dental bleaching has become one of the most common requests from patients, due to the ease of the procedure, minimal invasiveness, and immediate effectiveness in improving the esthetic aspect of the patients’ smile [3,4]. Manufacturers have introduced a range of bleaching products to the market, comprising different active agents and concentrations, which are intended for self-application in a domestic setting or for in-office professional use [5,6]. The most common bleaching agent used is hydrogen peroxide (H_2_O_2_), also delivered in the form of carbamide peroxide (CH_6_N_2_O_3_). Due to their low molecular weight, peroxides diffuse into enamel and dentine. As it diffuses through enamel prisms, hydrogen peroxide (HP) dissociates into peroxide anion (HO_2_^-^) and oxygen free radicals, collectively known as reactive oxygen species (ROS). ROS initiate an oxidation reaction of pigmented organic molecules, resulting in the formation of colorless inorganic compounds and a consequent whitening effect [5,7]. Carbamide peroxide (CP) only becomes active through a chemical reaction, dissociating approximately into 30% HP and 70% urea [8]. In general, low-concentration gels (HP 4% to 22%) are designed for at-home whitening procedures, as multiple applications are typically necessary, and high-concentration gels (HP 25% to 40%) are employed for professional in-office techniques [9].

The potential adverse effects of the use of HP have raised concerns. Increased hypersensitivity and microscopic changes to the tooth surface, resulting in reduced hardness and increased porosity and surface roughness, are reported [10,11,12,13]. The whitening effectiveness and potential adverse effects seem to depend upon the concentration, application protocol, diffusion capacity, and baseline pH of the bleaching solution [14,15,16]. Current evidence indicates that neutral or alkaline pH gels have comparable whitening effects to acidic gels, with reduced risk of dentin hypersensitivity and surface alterations [17,18,19]. However, more recent evidence suggests that the maintenance of pH levels during the bleaching process impacts surface alterations of the enamel [8,20,21].

This in vitro study aims to compare the whitening effectiveness, enamel surface roughness, and pH fluctuations after the procedure of three neutral bleaching agents on human extracted teeth. The null hypotheses (H_0_) to be tested were as follows: (I) all the bleaching agents have the same whitening effectiveness; (II) there are no differences among the bleaching agents in the pH variations recorded after the procedure; (III) there are no differences in the surface roughness changes.

## 2. Materials and Methods

### 2.1. Sample Selection and Preparation

The minimum sample size required to detect significant differences with a power of 80%, an effect size of 0.5, and an alpha error of 0.05 was determined using G*Power 3.1. A sample of 42 specimens (*n* = 14 for each group) was calculated. A pool of extracted human molars from an anonymized biobank was selected for this study. After extraction, the teeth were cleaned of soft tissues debris by gently scraping the external surface with periodontal instruments. The teeth were then stored in distilled water (≤1 month). Samples were inspected under a 10× optical microscope (OPMI Pico, Carl Zeiss Meditec Inc., Jena, Germany) to exclude caries, cracks, fractures, or restorations, and a total of 21 healthy teeth (42 specimens) were finally included.

**Figure 1:**
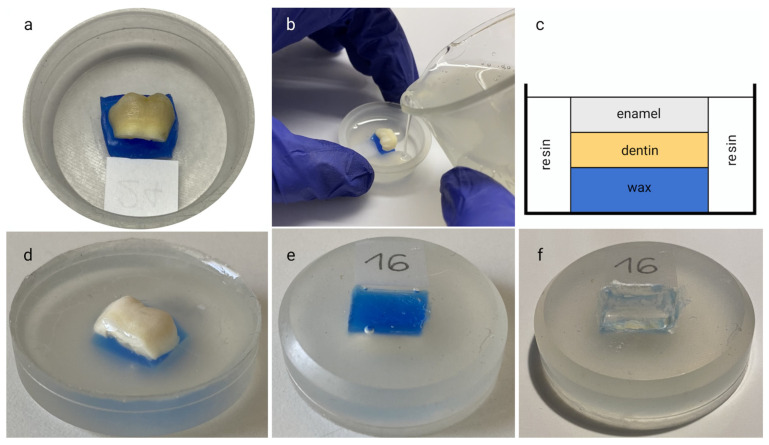
Sample preparation: (**a**) specimen placed in the plastic container with the wax lying on the bottom; (**b**) pouring of the transparent self-curing epoxy resin into the plastic mold (**c**) schematic illustrations of the sample; (**d**) specimen embedded in the cured resin after removal of the plastic container; (**e**) view of the waxed dentin surface of the specimen; (**f**) view of the dentin surface of the specimen after removal of the wax.

A high-speed fissure bur (size 701; Komet USA LLC, Rock Hill, SC, USA) was used to separate the crown from the root, at the level of the cement–enamel junction. The crown was split to obtain a buccal and a lingual surface for each tooth. The specimens obtained (*n* = 42) were then immersed for 7 days in Hanks’ balanced salt solution (HBSS; Sigma Aldrich, St. Louis, MO, USA). All specimens were dried with an air syringe and embedded in resin (Figure 1). The dentin surface was covered with dental impression wax (Zeta, Novi Ligure, Italy) and the specimens were placed in cylindrical plastic containers (30 × 10 mm), with the enamel surface placed in an upwards direction. Transparent self-curing epoxy resin (Hard Rock 554, Remet, Bologna, Italy) was poured into the molds (cure = 7 days). The plastic molds were then removed, and the dentin surfaces were exposed by scraping out the wax with a manual excavator, allowing for improved penetration of the staining solution. The samples were randomly divided into three groups, based on the bleaching agent.

### 2.2. Staining Procedure

**Figure 2:**
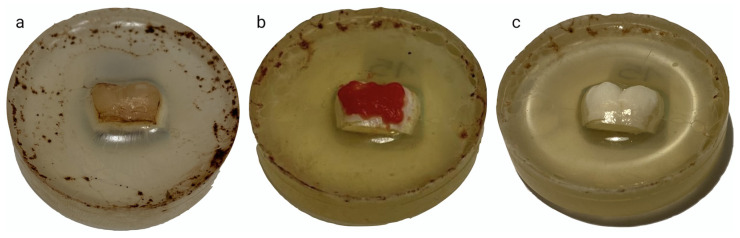
(**a**) Specimens after immersion in staining solution; (**b**) during bleaching treatment; (**c**) after bleaching treatment.

The samples were immersed in a staining solution of black tea obtained by placing 12 tea bags (Lipton Yellow Label Classic) in 1.5 L of boiling water for 25 days, as indicated by the manufacturer. The solution was stirred once a day to prevent its precipitation and the black tea solution was replaced three times throughout the period of staining. The samples were rinsed under running water to remove excess solution and dried with an air syringe (Figure 2).

### 2.3. Bleaching Procedure

Each bleaching agent was applied to the specimens according to the manufacturers’ recommendations.

The HP 40% group (*n* = 14) was treated with Opalescence Boost gel (Ultradent Italia, Milano, Italy), containing HP 40%, potassium nitrate, and sodium fluoride and having a neutral pH (7.0). The gel, intended for professional use, was applied and left in contact with the enamel surface for 20 min and then thoroughly cleansed with water. This procedure was repeated three times.

The HP 35% group (*n* = 14) was treated with Ena White Power gel (Micerium s.p.a, Avegno, Italy), containing HP 35%, potassium nitrate, and sodium fluoride and having a neutral pH (7.3) The gel, intended for professional use, was applied for 10 min and then thoroughly cleansed with water. This procedure was repeated three times.

The CP 16% group (*n* = 14) was treated with White Dental Beauty gel (Optident, Ilkley, UK), containing 16% CP (corresponding to HP 5.8%), potassium nitrate (0.11%), and sodium fluoride (1100 ppm) and having a neutral pH (6.5). This product is designed for a possible hybrid professional/at-home protocol and can also be used for multiple at-home applications. The gel was applied only once for 90 min. Finally, all the samples were rinsed under running water and dried with an air syringe (Figure 2).

### 2.4. Color Change Evaluation

The color of the samples was assessed using a portable digital spectrophotometer (Easyshade V, VITA, Bad Säckingen, Germany) before (T0) and after immersion in black tea (T1) and after the whitening procedure (T2). Color changes were recorded according to the CIE L*a*b* coordinate system, as defined by the International Commission on Illumination (CIE) [22,23]; L* indicates brightness, C* indicates chroma, h° indicates hue, and a* and b* indicate color coordinates ranging between green and red and between blue and yellow, respectively. A single measurement was taken at the center of the sample (approximately), placing the tip of the device (0.5 mm in diameter) perpendicularly in contact with the surface [24]. To ensure the accuracy and reliability of data, the spectrophotometer was calibrated using the calibration block provided with the device, at the beginning of each measurement session [25]. The color differences between time points were then determined by calculating ΔE_00_ (CIEDE2000 system, 2001), using the equations provided by Sharma et al. [26,27,28,29].

### 2.5. pH Analysis

A portable high-precision pH-meter (pH 6, XS Instruments, Carpi, Italy) was used to measure the pH stability. The pH values were measured on gels at T1 and T2 (before removing the agent from the sample surface).

### 2.6. Surface Roughness Evaluation

**Figure 3:**
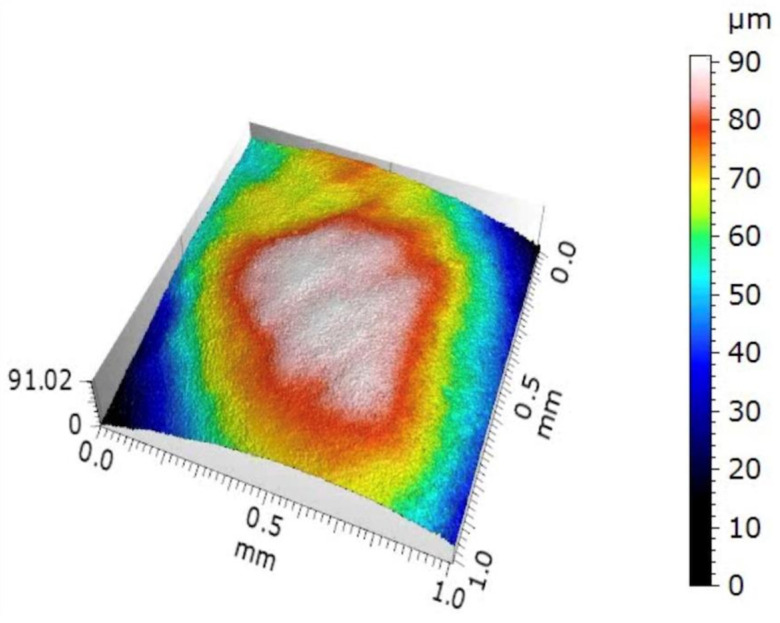
Tridimensional image of a sample obtained using MountainsMap 7.4 software for measuring area surface roughness (Sa) through optical profilometry.

Surface roughness (Sa) was measured at T1 and T2 to observe any changes in the enamel structure following the application of the bleaching gels. Sa was evaluated using an optical microscope (Eclipse LV150N, Nikon, Tokyo, Japan) equipped with an automated x–y–z table and a structured illumination camera (Confovis, Jena, Germany). The analysis is based on the “structured illumination microscopy” (SIM) measurement technology, which can be used as a profilometer. The system provides a 3D image of the specimen surface, which is processed using the MountainsMap 7.4 software (Digital Surf, Besançon, France) to obtain roughness values (Figure 3). Measurements were carried out using an objective with 10× magnification. The profiles were filtered using a robust Gaussian filter with a 25 μm cut-off length to separate the large-scale waviness of the tooth surface from the small-scale roughness. The arithmetic mean of the heights in the experimental area A (Sa) was considered as the roughness value, according to ISO 25178-2:2021 for Geometrical Product Specifications (GPS) [30]. Two measurements were taken for each sample (T1 and T2) and an average was calculated.

### 2.7. Statistical Analysis

All data were recorded in Excel datasheets (Microsoft Office, Redmond, WA, USA) and mean and standard deviations were calculated. The normal distribution of data was evaluated with the Shapiro–Wilk test. One-way analysis of variance (ANOVA) followed by Bonferroni’s post hoc test or Kruskal–Wallis followed by Dunn’s post hoc tests was chosen to compare the differences between groups according to the type of distribution. The *t*-test for paired samples was used to compare the surface roughness data within groups at T1 and T2. The level of significance was set at a *p*-value ≤ 0.05. Stata 11.2 (StataCorp LLC, College Station, TX, USA) was used for the analyses.

## 3. Results

### 3.1. Color Change Evaluation

The results of the color changes throughout the study are shown in Table 1. All the groups of specimens showed similar levels of color changes between T1 and T2 (not statistically significant). At T2, the color change of CP 16% was significantly lower compared to HP 40% (*p* = 0.004) and HP 35% (*p* = 0.001).

### 3.2. pH Analysis

The results of pH analysis are presented in Table 2. The neutral pH of the bleaching agents was confirmed at T1. At T2, a mild increase in pH values of both HP 35% (+0.26) and CP 16% (+0.03) was observed; there was a statistically significant difference between groups (*p* < 0.05). A slight pH decrease (−0.41) was recorded in HP 40%; significantly different from HP 35% only (*p* < 0.05).

### 3.3. Surface Roughness Evaluation

Sa values at T1 and T2 are shown in Table 3. At T1, Sa values were comparable among groups and decreased slightly in all groups at T2 (no significant difference between groups). Within the groups, Sa values at T2 were significantly lower in the HP 40% group (*p* = 0.02) only.

## 4. Discussion

The effectiveness of bleaching products has traditionally been related to the concentration of peroxides present as the active agent. However, research has also investigated the influence of other factors such as pH and the addition of chemical conditioners, with the aim of enhancing the whitening results and minimizing potential side effects [31,32].

The first null hypothesis of this study was rejected, since the two bleaching agents with slightly different HP concentrations (35% and 40% HP) showed comparable results in terms of whitening effect. These results have been confirmed in similar studies [5,11]. The whitening effect of the CP 16% gel (HP 5.8%) was significantly lower than that of HP 30% and HP 40% according to spectrophotometer analyses. As CP 16% is intended for an initial in-office session, with possible additional applications at home, our study design, with a single in-office application, may underestimate the overall clinical whitening effect [33].

A variety of color assessment techniques can be employed, including subjective methods such as visual analysis by comparison with a standard shade guide, and other objective methods, such as the use of colorimeters and image analyses [24,34]. However, this study employed the spectrophotometric color assessment method, as there are advantages in accuracy and reproducibility in an experimental setting [35]. It should be noted, however, that significant differences identified through the spectrophotometric technique may not necessarily correspond to differences discernible to the human eye, and thus may not be deemed clinically relevant [36,37]. Various studies have indicated that the ΔE required for observers to clinically perceive a color difference is 1 to 3 ΔE units, approximately [38,39]. This reveals the difficulty of identifying a correspondence between experimental ΔE values and clinical perception of differently defined colors [38,39].

Peroxides act by dissociating into hydroxyl radicals, such as HO_2_^-^ [33]. CP is more stable and results in a prolonged and slower active whitening process when compared to HP [31]. Products with higher dissociation rates rapidly deplete their effect and typically require an early replacement of the material and repeated applications, whereas products with a lower dissociation rate exhibit a prolonged and slower active whitening process, requiring fewer applications [8]. Overall, HP concentration is not the only variable relevant to whitening effectiveness, as the pH of the solution, duration and number of applications, and use of enhancing agents during the procedure such as laser or UV light exposure also influence the outcome [40,41,42,43].

A study by Ozdemir et al. compared different application times of HP 40% on enamel in terms of whitening effect, microhardness, and surface roughness. The authors reported that the optimal time to minimize surface changes and for the best whitening efficacy was the intermediate 40-min application. However, the authors reported an effective whitening effect and no significant increase in surface roughness in all application groups [19]. Similarly, recent studies found a significant reduction in microhardness and an increase in surface roughness of enamel after bleaching compared to baseline values, with acidic pH and regardless of the concentration of HP used [13,21].

When examining the whitening effectiveness in relation to pH, a number of studies found an increase in the speed of the reaction between pH 8.0 and pH 9.0, thereby suggesting a greater effectiveness for gel with an alkaline pH [20,44]. However, none of these studies examined potential shifts from the initial pH over the course of application, which is an important factor in determining the interaction of a bleaching agent with the tooth substrate. In fact, the change in enamel physical properties is more likely due to the demineralization effects caused by the diffusion of HP and the acidic pH of the bleaching gel than to the effect of the peroxide per se [13,45]. The effect of a low pH on the tooth has also been associated with a more pronounced penetration of HP ions through the tooth structure, towards the pulp, causing increased sensitivity [17,46]. In the present study, we also examined pH variations and compared them between groups. As for homogeneity of pH variation, the second null hypothesis was rejected, since CP 16% was substantially stable, while HP 35% showed a mild pH increase and HP 40% was less stable. The greater stability exhibited by CP 16% is consistent with CP being a more stable compound than HP-based products [31].

Although some existing literature has indicated a general tendency to progressive acidification of higher-HP-content gels throughout the application, other studies have demonstrated that acidification or alkalinization is not dependent on HP concentration [8,47]. Whilst HP 40% and HP 35% are both highly concentrated, HP 40% showed a significant tendency to acidification and HP 35% to alkalinization. CP 16%, a low-concentrated product, showed only a mild pH increase.

The third null hypothesis was rejected, since all groups showed a slight reduction of the enamel Sa, with a statistically significant change in HP 40%. The Sa of HP 35% changed slightly but it was applied for a shorter recommended time compared to HP 40%. This suggests that a longer application time could potentially sustain the observed tendency towards further surface changes. The results of the present study appear to be in conflict with the majority of similar studies, which have documented either a slight increase or no change in enamel surface roughness following the application of bleaching agents [5,11,13,19,48,49,50].

Some discrepancies in results are attributable to differences in study designs. Some studies perform enamel polishing procedures [13,19,50]. Conversely, our study aimed to preserve the natural enamel surface roughness, and specimens were not polished. Additionally, the samples were subjected to a controlled staining procedure at T1, and some residual solution may have remained on the tooth surface. In comparison to other studies, our sample exhibited a greater baseline surface roughness, which resulted in a general tendency towards roughness reduction due to the removal of surface irregularities caused by the bleaching treatment. Indeed, chemical agents may reduce the roughness of an uneven surface by selectively targeting the ridges, whereas they may conversely increase the roughness of a smooth surface where ridges are absent because of previous polishing. Further, differing methods of surface roughness assessment are used; most other studies used a profile (line) roughness parameter obtained through a stylus contact profilometer method, whereas we chose an area roughness parameter (i.e., Sa), obtained through optical 3D profilometry [5,9,11]. Although profile roughness (Ra) is more commonly used due to its simplicity and cost-effectiveness, surface roughness (Sa) is a more sensitive method, which provides more consistent measurements and is able to detect more mild changes [51]. In addition, a number of studies used bovine incisors as enamel samples, which are considered similar to human enamel in mineral composition and physical properties, whereas we used human extracted molars [20,21,50,52].

Roughness changes can influence the interaction between the enamel surface and the local environment. It has been demonstrated that a rougher surface can promote bacterial adhesion and plaque maturation, as well as augmented adsorption and penetration of pigmented molecules [53,54]. However, changes observed in the present study were overall smaller than the threshold considered to be critical for plaque retention (i.e., Ra of 0.2 µm) [55]

Changes in the surface roughness can be influenced by multiple factors, including pH, duration of the application, concentration of the bleaching agent, and viscosity of the mixture [5,21]. High concentrations of HP have further detrimental effects on the enamel surface if the pH of the gel is acidic, creating an environment that favors erosion and demineralization [13]. Some studies have reported that greater longer application times result in greater impact on surface changes when compared to the concentration of the bleaching agent [56,57,58]. Additionally, according to some authors, the viscosity of the mixture does not influence bleaching effectiveness, but low viscosity promotes penetration of HP into the tooth structure, causing dissolution both at the level of enamel prisms and interprismatic area, influencing surface roughness [48,59,60]. Thus, a higher concentrations of HP in association with lower-viscosity products could result in a greater penetration of the perhydroxyl anion and more pronounced surface changes [61].

Overall, these findings suggest that the effectiveness of bleaching products does not depend solely on their pH and corroborate the evidence from other studies indicating that neutral and alkaline mixtures can be equally or even more effective than acidic ones and limit possible adverse effects associated with acidic products [8,45]. Alkaline or neutral gels have shown a greater release of hydroxyl radicals, combined with a lower diffusion into the dental structure towards the pulp chamber, possibly leading to hypersensitivity [17,18].

The pH of the mixture can be influenced by the addition of various components. For instance the acidic and ionic thickening agent carbopol has been reported to affect enamel roughness more than natrosol, a cellulose-based non-ionic thickening agent [62].

Also, different conditioners with different pH values, such as sodium hydroxide (NaOH), sodium bicarbonate (NaHCO_3_), sodium carbonate (Na_2_CO_3_), and potassium bicarbonate (KHCO_3_), can influence the whitening efficacy [63]. For example, according to the information available from the manufacturers, the amounts of potassium nitrate and sodium fluoride were not available for all gels. Further, HP 35% also contains sodium hydroxide. Thus, the specific and complex chemical composition of the tested products presumably plays an important role in their interactions with the oral environment and may explain the differences observed in the outcomes.

The main limitations of this study are related to its in vitro design. It was not possible to ascertain the effect of pre-existing conditions, such as dental age or possible prior interventions, due to the random selection of the sample teeth, and this may have influenced the results. Furthermore, the absence of the pulp chamber, as well as the absence of pulp pressure, which can affect the penetration of the mixture through the tooth structure, precludes the assessment of the actual effect of bleaching agents on pulp tissue and the correlation of the tested parameters to adverse effects, such as increased sensitivity [13]. Additionally, the presence of saliva in a real in vivo setting, with its buffering and remineralizing functions, plays an important role in counteracting the potential adverse effects of bleaching treatments [64]. Lastly, as previously mentioned, the experimental setting did not allow for the compensation of the heterogeneous application times recommended for the different products, which may have biased the results.

Future studies should focus on the correlation in vivo among specific product composition, changes, effectiveness, and increased sensitivity. Also, a real-time monitoring of pH variation during the application may provide further insight on the dissociation rates of the bleaching products and on their effects on dental structures.

## 5. Conclusions

Among the bleaching agents tested in our study, high-HP-concentration agents seem to increase whitening effectiveness, while a low-CP-concentration agent is less effective. Within the limitations of this study, HP concentration did not appear to be directly related to pH changes and the bleaching effectiveness was not pH dependent. Conversely, enamel surface modifications were more pronounced with higher-HP-concentration agents, which also exhibited a greater tendency to pH instability following the procedure.

## Figures and Tables

**Table 1:** Color changes measured using CIEDE2000 system (ΔE_00_ ± standard deviation) of the samples before and after staining procedure (T0–T1) and before and after bleaching treatment (T1–T2), according to the type of treatment administered.

Treatment Groups	Color Change	
	T0–T1	T1–T2
	ΔE00 ± SD	ΔE00 ± SD
HP 40% (*n* = 14)	22.91 ± 13.05 ^a^	25.32 ± 12.77 ^a^
HP 35% (*n* = 14)	24.94 ± 13.60 ^a^	27.64 ± 12.95 ^a^
CP 16% (*n* = 14)	20.87 ± 6.55 ^a^	14.78 ± 7.99 ^b^

T0: before immersion in black tea; T1: after immersion in black tea; T2: after bleaching treatment. SD: standard deviation. Same superscript letter indicates a *p* > 0.05 between groups (Kruskal–Wallis test); T1–T2: HP 40% vs. HP 35% *p* > 0.05, HP 40% vs. CP 16% *p* = 0.004, HP 35% vs. CP 16% *p* = 0.001 (Dunn’s test).

**Table 2:** pH variation (ΔpH ± standard deviation) of the bleaching solution before application and at the end of the application, according to the type of treatment administered.

Treatment Groups	pH Before Bleaching (T1)	pH After Bleaching (T2)	ΔpH
	Mean ± SD	Mean ± SD	Mean ± SD
HP 40% (*n* = 14)	7.01 ± 0.00	6.60 ± 0.55	−0.41 ± 0.55 ^a^
HP 35% (*n* = 14)	7.08 ± 0.00	7.34 ± 0.03	0.26 ± 0.03 ^b^
CP 16% (*n* = 14)	6.90 ± 0.00	6.93 ± 0.02	0.03 ± 0.02 ^a^

SD: standard deviation. Same superscript letter indicates a *p* > 0.05 between groups (Kruskal–Wallis test); ΔpH HP 40% vs. HP 35% *p* = 0.0002, HP 40% vs. CP 16% *p* = 0.43, HP 35% vs. CP 16% *p* = 0.0003.

**Table 3:** Surface roughness values (Sa ± standard deviation) of the samples before and after bleaching treatment, according to the type of treatment administered.

Treatment Groups	Surface Roughness		
	Before Bleaching	After Bleaching	
	Sa ± SD [μm]	Sa ± SD [μm]	*p*
HP 40% (*n* = 14)	0.53 ± 0.10 ^a^	0.46 ± 0.08 ^a^	0.02 *
HP 35% (*n* = 14)	0.58 ± 0.20 ^a^	0.48 ± 0.10 ^a^	0.067
CP 16% (*n* = 14)	0.47 ± 0.11 ^a^	0.44 ± 0.08 ^a^	0.29

SD: standard deviation. Same superscript letter indicates a *p* > 0.05 between groups (one-way ANOVA test); * indicates a significant difference within groups (*t*-test for paired samples).

## Data Availability

Any additional data supporting these findings are available from the corresponding author upon reasonable request.
